# Complete genome sequence of the rumen bacterium *Butyrivibrio fibrisolvens* D1^T^

**DOI:** 10.1128/mra.00267-24

**Published:** 2024-04-23

**Authors:** Nikola Palevich, Faith P. Palevich, Graeme T. Attwood, William J. Kelly

**Affiliations:** 1 Grasslands Research Centre, AgResearch Ltd., Palmerston North, New Zealand; 2 Hopkirk Research Institute, AgResearch Ltd., Palmerston North, New Zealand; Portland State University, Portland, Oregon, USA

**Keywords:** *Butyrivibrio fibrisolvens*, bacteria, rumen, genome, plasmids

## Abstract

*Butyrivibrio* are anaerobic bacteria and members of the family *Lachnospiraceae* with important roles in fiber digestion in both animals and humans. This report describes the complete genome of *Butyrivibrio fibrisolvens* type strain D1^T^ (DSM 3071) consisting of a chromosome (CP146963), megaplasmid (pNP243), and small plasmid (pNP21).

## ANNOUNCEMENT

The anaerobic butyrate-producing bacterium *Butyrivibrio fibrisolvens* D1^T^ (ATCC 19171 and DSM 3071) is the type species of the genus *Butyrivibrio* within the family *Lachnospiraceae* (1). *B. fibrisolvens* D1^T^ is Gram positive but stains Gram negative (2) and can ferment a wide range of carbohydrates, including xylan, pectin, and starch, with the production of H_2_, CO_2_, butyrate, formate, and lactate. D1^T^ was originally isolated from bovine rumen contents inoculated into 40% rumen fluid-glucose-cellobiose agar medium roll tubes (3). The available draft genome of D1^T^ (GCA_900129945) is the sole member of *Butyrivibrio fibrisolvens* species group in the Genome Taxonomy Database Toolkit (GTDB-Tk), while 10 other isolate genomes cluster as *Butyrivibrio fibrisolvens_C* (4, 5). This report describes the complete genome of D1^T^ consisting of a 4,671,138 bp circular chromosome (GenBank accession number CP146963), a 242,710 bp megaplasmid (pNP243 and CP146964), and a 20,499 bp small plasmid (pNP21 and CP146965).

For genome sequencing, strain D1^T^ was acquired from the Leibniz Institute DSMZ (German Collection of Microorganisms and Cell Cultures), and pure cultures were passaged once before isolating the genomic DNA. To prepare the genomic DNA, strain D1^T^ was cultured anaerobically at 39°C in 50 mL RM02 medium supplemented with cellobiose, casamino acids, Bacto Peptone, and yeast extract (6). Genomic DNA was extracted using Wizard HMW DNA Extraction Kit (Promega), including RNase treatment, following the manufacturer’s instructions. DNA quantity, quality, and integrity were assessed using the High Sensitivity DNA LabChip Kit on the Bioanalyzer 2100 (Agilent Technologies). The 16S rRNA gene was amplified using AmpliTaq Gold 360 master mix (Applied Biosystems) with M13 forward (5′-CCCAGTCACGACGTTGTAAAACG-3′) and reverse (5′-AGCGGATAACAATTTCACACAGG-3′) primers and sequenced on an ABI 3730 DNA system (Applied Biosystems). Strain identity was confirmed (GenBank accession no. U41172) via alignment to the NCBI 16S rRNA database using BLAST v2.9.0 (7).

Approximately 23 µg of fresh genomic DNA was transferred into GenTegra DNA tubes (GTD2003-S) and sequenced using Illumina and Oxford Nanopore Technologies (ONT) platforms. For Illumina, 150-bp paired-end reads were sequenced using VAHTS Universal Plus DNA Library Prep Kit v2 (ND617-C3-02; Vazyme) on the NovaSeq6000 instrument. Raw sequencing data were demultiplexed and quality assessed using bcl2fastq v2.20 with ASCII Qscore offset 33 producing a mean Phred quality score of 36.4. Prior to library preparation for ONT sequencing, g-TUBEs (Covaris) were used to fragment the genomic DNA and select for fragment sizes of >10 kb. Long-read libraries were prepared using the ONT ligation sequencing kit v14 (SQK-LSK114) and multiplexed using the native barcoding kit (EXP-NBD104). ONT sequencing was performed on a PromethION flow cell (FLO-PRO002) using a PromethION 48 instrument with MinKNOW v22.8.9 used for data acquisition. Base calling, demultiplexing, adapter trimming, and quality filtering (*Q* > 7; sequence length > 1,000 bp) of raw sequences were performed by using Guppy v6.5.7. Quality-filtered ONT reads were assembled *de novo* using Canu v2.2 (8) and polished with alignments of the short-read data using Racon v1.4.3 (9). Quality and completeness were evaluated using CheckM v1.2.2 (10), with annotation performed using NCBI’s Prokaryotic Genome Annotation Pipeline v6.6 (11). Taxonomic assignment was based on 16S rRNA gene sequences and using the Genome Taxonomy Database Toolkit (GTDB-Tk) v2.3.0 (12). Default parameters were used for all instruments and software. The sequencing data metrics and genome features are summarized in Table 1.

**TABLE 1:** *Butyrivibrio fibrisolvens* D1^T^ genomic features.

**Metadata**	
Strain identifiers	D1, DSM 3071, ATCC 19171 (1)
Isolation source	Bovine rumen
**Genome project information**	
BioProject accession ID	PRJNA1083737
BioSample ID	SAMN40258195
GenBank accession numbers	CP146963, CP146964, CP146965
Assembly ID	GCA_037113525
**ONT data**	
SRA accession|Run IDs	SRX23834893 |SRR28223538
No. of raw reads	1,384,008,505
No. of filtered reads	228,208
*N*_50_ of filtered reads (bp)	10,204
**Illumina data**	
SRA accession|Run IDs	SRX23834892| SRR28223539
No. of raw reads	2,008,639,500
No. of filtered reads	6,695,465
**Genome assembly statistics**	
Assembly size (bp)	4,934,347
Genome coverage (×)	295
G + C content (%)	39.5
No. of contigs	3
Contig *N*_50_ (bp)	4,671,138
CheckM completeness|contamination (%)	93.7|8.29
**Genome annotations**	
No. of genes	4,208
No. of PCGs^*a*^	4,110
No. of rRNAs	6
No. of tRNAs	53
**Taxonomy**	
NCBI taxon ID	1121131
GTDB representative	Butyrivibrio fibrisolvens

^*a*^ PCGs, protein coding sequences.

## Data Availability

The complete genome sequence and associated data for *Butyrivibrio fibrisolvens* D1^T^ have been deposited under the GenBank BioProject accession number PRJNA1083737. Table 1 details information regarding GenBank, BioSample, Assembly, and Sequence Read Archive (SRA) accession numbers.
